# Case report: A patient coinfected by *Borrelia burgdorferi* sensu lato and spotted fever group Rickettsiae in Urumqi, China

**DOI:** 10.1097/MD.0000000000017977

**Published:** 2019-11-15

**Authors:** Yi Jiang, Xuexia Hou, Lin Zhang, Yuhui Tan, Chen Lu, Dong Xiao, Hongyan Li, Qin Hao, Kanglin Wan

**Affiliations:** a National Institute for Communicable Disease Control and Prevention, Chinese Center for Disease Control and Prevention / State Key Laboratory for Infectious Disease Prevention and Control, Beijing; b Collaborative Innovation Center for Diagnosis and Treatment of Infectious Diseases, Hangzhou; c Department of Neurology, Xinjiang Uygur Autonomous Region Hospital, Urumqi, PR China.

**Keywords:** *Borrelia burgdorferi* sl, co-infection, spotted fever group Rickettsiae

## Abstract

**Rationale:**

Both *Borrelia burgdorferi* sensu lato and spotted fever group Rickettsiae (SFGR) are pathogens carried by ticks. There is a possibility of co-infection with these tick-borne diseases.

**Patient concerns:**

Male patient, 63 years-of-age, admitted to hospital with skin rash presenting for 1 week and fever with cough and expectoration for 3 days before admission.

**Diagnoses:**

We diagnosed that the patient was co-infected by *B burgdorferi* sl and SFGR using laboratory test results and the patient's clinical manifestations.

**Interventions:**

The patient started therapy with oral minocycline, then levofloxacin by intravenous injection for SFGR. Meanwhile, he was treated with penicillin G sodium, cefoperazone sulbactam sodium and ceftriaxone by intravenous injection for *B burgdorferi* sl.

**Outcomes:**

After the patient was in stable condition, he was discharged from hospital.

**Lessons:**

This case report highlights the possibility of co-infection by 2 tick-borne diseases in Urumqi, Xinjiang Uygur Autonomous Region, China. The antibiotic therapy should be based on the detection of pathogenic bacteria, and the different susceptibilities of co-infecting bacteria should be considered.

## 1 Introduction

Lyme disease is a multisystem disease caused by strains of the spirochete *Borrelia burgdorferi*, a zoonotic pathogen transmitted by ticks. Tick-borne rickettsioses are caused by obligate intracellular bacteria of the spotted fever group Rickettsiae (SFGR). More than 800 species of tick have been found worldwide,^[1]^ and around 110 species have been recorded in China.^[2]^ Patients bitten by an infected tick can develop symptoms such as anaphylaxis, ulcers or inflammation. In addition, many species of tick can carry and transmit pathogens, including viruses, protozoans, and bacteria including spirochetes and Rickettsiae.^[3]^ Co-infection with Lyme disease and other pathogens such as *Anaplasma phagocytophilum* or *Babesia* has frequently been reported around the world.^[4–8]^ However, in China, there are only a few reports of co-infection of patients with *B burgdorferi* and other pathogens.^[9,10]^ This report describes the case of a patient co-infected by *B burgdorferi* sensu lato (*B burgdorferi* sl) and SFGR in Urumqi, China.

## 2 Case history

A 63-year-old man was admitted to the Department of Neurology, Xinjiang Uygur Autonomous Region Hospital, Urumqi, China, in May 2017 with skin rash presenting for 1 week and fever with cough and expectoration for 3 days before admission.

**Figure 1:**
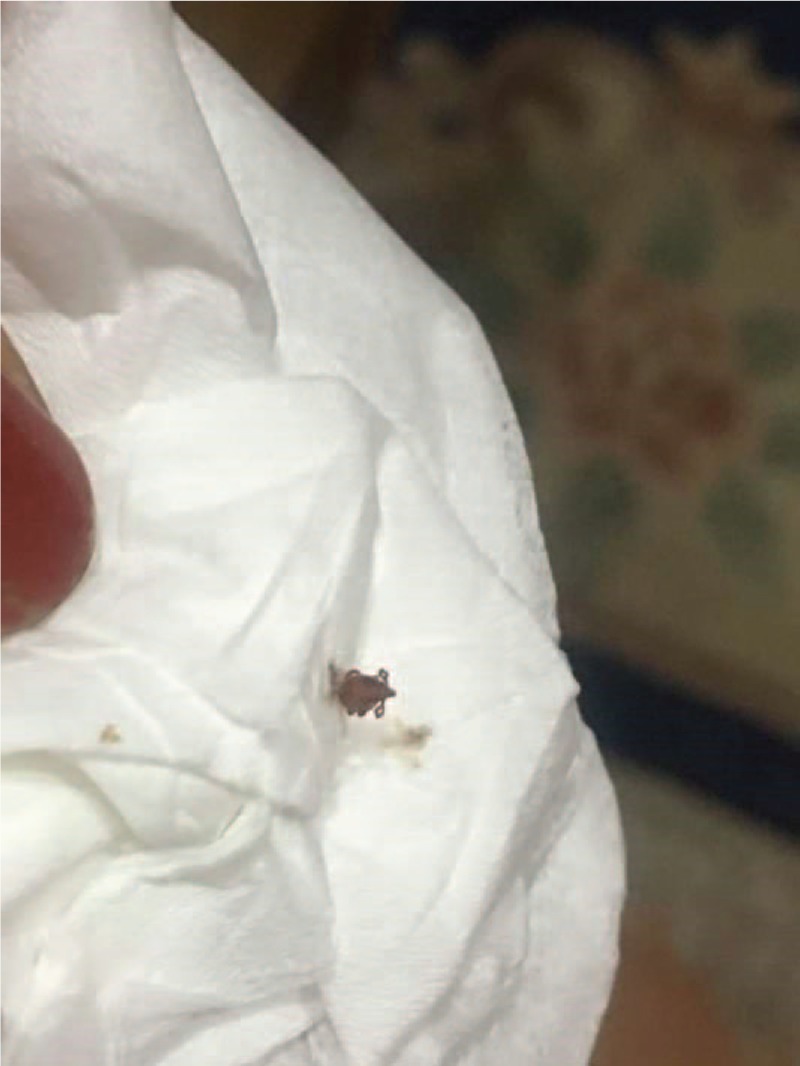
The *Ixodes persulcatus* that bit the patient.

**Figure 2:**
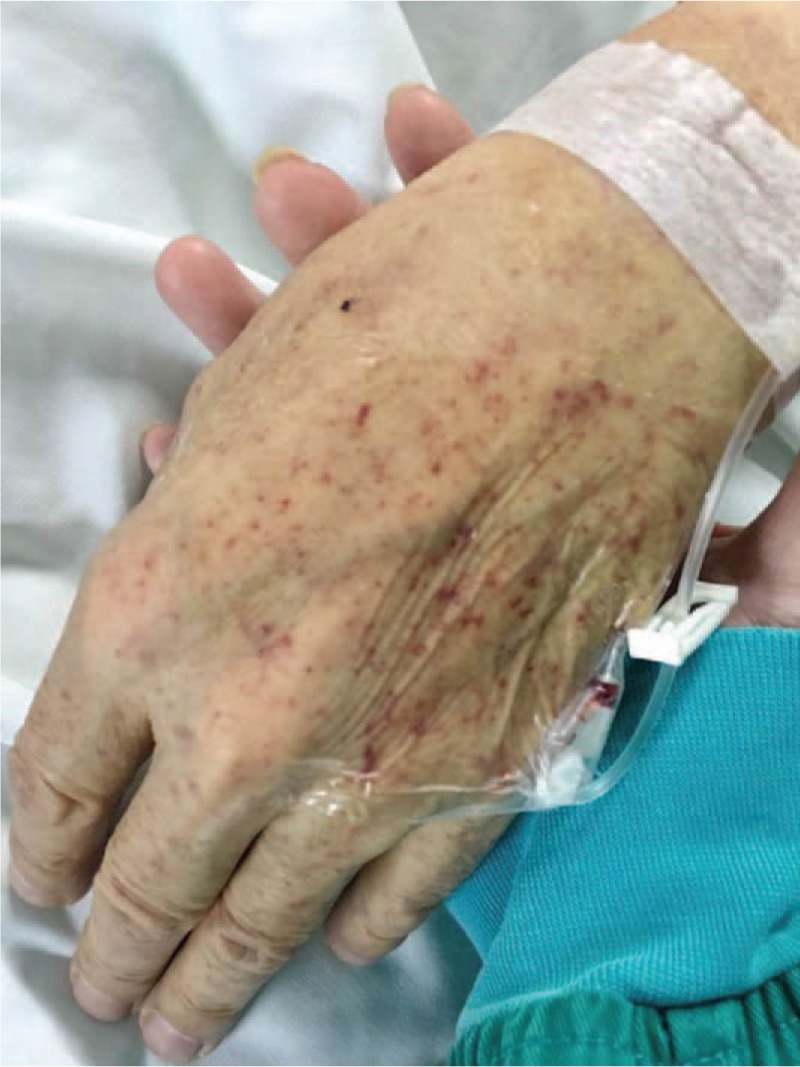
The rash on the patient's hand.

He recalled that he was bitten by a tick on the right earlobe 1 week before admission. Two days after that, he had a severe stabbing headache, which was aggravated at night. The next day, he had fever as high as 38.5°C, body aches and fatigue. Two days later, he had a generalized rash distributed on the trunk and limbs (diffuse symmetrical red hill rash which faded when pressed). The patient also suffered conjunctival hyperemia, cough, expectoration (thick-yellow sputum), and shortness of breath. Figure 1 shows the *Ixodes persulcatus* (a type of tick) that bit the patient. The rash on the patient's hand is displayed in Figure 2.

Physical examination on hospital admission revealed a temperature of 39°C, blood pressure of 100/60 mmHg, and a pulse rate of 97 beats per minute. The patient had clubbed toe. The rest of the examination was in normal limits. Laboratory testing revealed high values for white blood cell count (13.94 × 10^9^/L), absolute neutrophil count (13.03 × 10^9^/L) and C-reactive protein (159.02 mg/L). The patient had low values for red blood cell count (3.92 × 10^9^/L), hemoglobin level (124 g/L), and lymphocyte count (0.39 × 10^9^/L). Creatinine and bilirubin were within normal limits. The levels of glutamic-pyruvic transaminase (64.00 U/L), glutamic-oxalacetic transaminase (92.00 U/L), and lactate dehydrogenase (400.00 U/L) were elevated.

We collected serum samples from the patient 2 days after hospital admission (in the acute stage) and 19 days after admission (in the recovery stage). Indirect immunofluorescence assay (IFA), western blotting (WB) and nested polymerase chain reaction (nested PCR), were performed to confirm *B burgdorferi* sl infection. Indirect IFA was carried out to confirm SFGR infection.

Serological and nested PCR tests were performed in the National Institute for Communicable Disease Control and Prevention, Chinese Center for Disease Control and Prevention. In the samples taken 2 days after hospital admission, serological tests for *B burgdorferi* sl were positive. The presence of IgM against specific antigens of *B burgdorferi* sl was determined by IFA and WB (criteria according to previous description^[11]^). Serological test for *Rickettsia* was also positive; the presence of IgG against specific antigens of SFGR was determined by indirect IFA (*Rickettsia* IgG antibody test kit, Focus Diagnostics, USA).

Nested PCR was performed using a Bio-Rad Thermal Cycler (Bio-Rad Laboratories, Inc, Hercules, CA) as previously described.^[12]^ The DNA of *B burgdorferi* sl was detected using nested PCR targeting rrf (5S)-rrl (23S) intergenic spacer rRNA. The minimum detectable concentration of *B burgdorferi* sl DNA by this method is 10^2^ copies. DNA from blood was extracted using a DNeasy Blood and Tissue Kit (Qiagen, USA) according to the manufacturer's instructions. A positive control (*B burgdorferi senso stricto* strain PD_91_) and a negative control (water) were used on every plate tested. The nested-PCR results were positive for *B burgdorferi* sl in samples taken from the patient 2 days after hospital admission.

Fourteen days after admission, the patient developed neurological symptoms such as acute cerebral syndrome, organic mental disorder and delirium. The patient had an abnormal electroencephalogram (EEG; the 2 hemispheres had low–middle amplitude 4 to 6 θ-waves, some low amplitude 12 to 20 Hz β-waves, and some scattered middle amplitude 2 to 3 Hz δ-waves. There were no obvious focal changes.)

Table 1 shows the results of laboratory tests for the patient. Based on clinical manifestations and laboratory test results, we diagnosed that the patient was co-infected by *B burgdorferi* sl and SFGR.

**Table 1:**
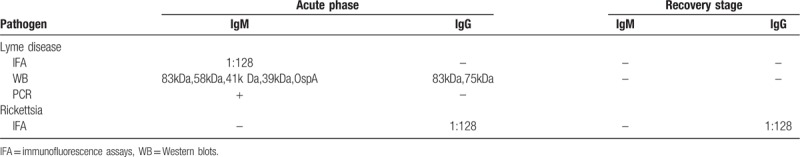
Laboratory tests of the patient coinfected with Lyme disease and rickettsioses.

The patient started therapy with oral minocycline (200 mg first time, then 100 mg twice daily) on day 2 and 3 after hospital admission. From day 3 to day 11, he was treated with levofloxacin by intravenous injection (500 mg, once daily). Meanwhile, the patient was treated with penicillin G sodium by intravenous injection (4 million U, every 6 hours) from day 2 to day 11. Cefoperazone sulbactam sodium by intravenous injection (4 g, every 12 hours) was administered to the patient from day 10 to day 15. On day 15, the rash on the patient completely subsided. From day 15 to day 17, the patient was treated with ceftriaxone^[13]^ by intravenous injection (2 g, every 12 hour) and oral administration of quetiapine fumarate tablets^[14]^ (12.5 mg, every night). From day 17, the patient's nervous system symptoms gradually reduced. Finally, the patient was treated with ceftriaxone by intravenous injection (2 g, every 12 hour) from day 17 to day 20.

Nineteen days after hospital admission, *B burgdorferi* sl serological and nested PCR tests were performed again and all the results were negative. However, serological tests for SFGR were positive. After the patient was in stable condition, he was discharged.

## 3 Discussion

Hard ticks (Ixodidae) have been identified as vectors of SFGR in humans.^[15,16]^*Ixodes* ticks are also the main vector of *B burgdorferi* sl, the causative agent of Lyme disease.^[4]^ The clinical symptoms of SFGR usually begin 4 to 10 days after a tick bite and vary depending on the *Rickettsia* species involved.^[17]^ Typical signs include fever, headache, malaise, muscle pain, rash, and local lymphadenopathy.^[18]^ For most SFGR, a characteristic skin lesion at the site of the tick bite, known as an eschar, may occur.^[18]^ Lyme disease can affect multiple systems of the body and produce a series of symptoms including erythema migrans, fever, arthritis, neurologic and heart damage, and may continue to develop severe chronic consequences such as neurological symptoms and disability.^[10]^ Our patient was bitten by a tick that is a vector of both SFGR and *B burgdorferi* sl In the early stage, the patient presented with skin rash, fever, and cough with expectoration. He exhibited a typical SFGR clinical picture, including a maculopapular rash and a black eschar at the site of the tick bite. In the later stage, he presented neurological symptoms, which were likely produced by *B burgdorferi* sl.^[19–21]^

Since culture or visualization of pathogens from patient specimens is difficult, diagnosis depends on recognition of a characteristic clinical picture with serologic confirmation. The current diagnosis for *B burgdorferi* sl usually includes enzyme-linked immunosorbent assay for screening and WB for confirmation.^[11]^ Clinical diagnostics for SGFR rely on the detection of antibodies against *Rickettsia* spp, which is the reference method.^[22]^ In our patient, in the acute phase, the presence of antibodies against specific antigens of *B burgdorferi* sl was determined by IFA and WB. Meanwhile, the patient had a strong antibody response to SFGR, demonstrated by IFA using *R conorii* antigen, confirming the diagnosis of SFGR. Nested PCR detection of *B burgdorferi* sl was also positive, further confirming infection with this pathogen.

*B burgdorferi* sl and SFGR co-infection occurs in *I ricinus* and Lyme borreliosis patients, or patients suspected of Lyme borreliosis, may indeed be exposed to both tick-borne pathogens. In the Netherlands, 25% of ticks infected by *B burgdorferi* sl were co-infected with *Rickettsia* spp.^[4]^ From 1987 to 1996, a serological survey of 22 provinces in China (including Xinjiang Uygur Autonomous Region) found that the positive rate of serum antibody to *B burgdorferi* sl in the population was 1.06% to 12.83%, and the average infection rate was 5.06%.^[23]^ There have been few seroepidemiological surveys of SFGR infection in the Chinese population. However, there are some reports of co-infection cases of *B burgdorferi* sl and SFGR in ticks.^[24]^ In 2012, there was a report of a cluster of three cases in Shandong Province of co-infection with Lyme disease and rickettsioses, which had closely associated epidemiological histories.^[10]^ Xinjiang Uygur Autonomous Region is one of natural foci of tick-borne diseases in China. This report might be the first case of co-infection by *B burgdorferi* sl and SFGR in Urumqi, the capital of Xinjiang Uygur Autonomous Region.

Various antibiotics have been used with success for the treatment of *B burgdorferi* sl and SFGR—minocycline and levofloxacin for SFGR,^[25]^ and penicillin G sodium, cefoperazone sulbactam sodium and ceftriaxone for *B burgdorferi* sl^[21]^ In our case, timely antibiotic administration may have shortened the symptomatic period and prevented the appearance of severe complications. Also, our patient was treated with ceftriaxone^[13]^ by intravenous injection and oral administration of quetiapine fumarate tablets^[14]^ to relieve nervous system symptoms.

This case report highlights the possibility of co-infection by 2 kinds of tick-borne disease in Urumqi, China. Antibiotic therapy should be based on the detection of pathogenic bacteria, and the different susceptibilities of co-infecting bacteria should be considered.

## Author contributions

**Conceptualization:** Qin Hao, Kanglin Wan.

**Data curation:** Yuhui Tan, Chen Lu, Dong Xiao, Hongyan Li.

**Investigation:** Xiaxue Hou, Lin Zhang.

**Writing – original draft:** Yi Jiang.
